# Diet and density dependent competition affect larval performance and oviposition site selection in the mosquito species *Aedes albopictus* (Diptera: Culicidae)

**DOI:** 10.1186/1756-3305-5-225

**Published:** 2012-10-08

**Authors:** Miho Yoshioka, Jannelle Couret, Frances Kim, Joseph McMillan, Thomas R Burkot, Ellen M Dotson, Uriel Kitron, Gonzalo M Vazquez-Prokopec

**Affiliations:** 1 Emory University, Georgia, Atlanta, USA; 2 Centers for Disease Control and Prevention, Atlanta, USA; 3 James Cook University, Cairns, Australia; 4 Fogarty International Center, National Institutes of Health, Bethesda, MD, USA; 5 Department of Environmental Studies, Emory University, Atlanta, GA, 30322, USA

**Keywords:** Habitat selection, Oviposition, Diet, Conspecific density, Mosquito

## Abstract

**Background:**

Oviposition-site choice is an essential component of the life history of all mosquito species. According to the oviposition-preference offspring-performance (P-P) hypothesis, if optimizing offspring performance and fitness ensures high overall reproductive fitness for a given species, the female should accurately assess details of the heterogeneous environment and lay her eggs preferentially in sites with conditions more suitable to offspring.

**Methods:**

We empirically tested the P-P hypothesis using the mosquito species *Aedes albopictus* by artificially manipulating two habitat conditions: diet (measured as mg of food added to a container) and conspecific density (CD; number of pre-existing larvae of the same species). Immature development (larval mortality, development time to pupation and time to emergence) and fitness (measured as wing length) were monitored from first instar through adult emergence using a factorial experimental design over two ascending gradients of diet (2.0, 3.6, 7.2 and 20 mg food/300 ml water) and CD (0, 20, 40 and 80 larvae/300 ml water). Treatments that exerted the most contrasting values of larval performance were recreated in a second experiment consisting of single-female oviposition site selection assay.

**Results:**

Development time decreased as food concentration increased, except from 7.2 mg to 20.0 mg (Two-Way CR ANOVA Post-Hoc test, *P > 0.1*). Development time decreased also as conspecific density increased from zero to 80 larvae (Two-Way CR ANOVA Post-Hoc test, *P < 0.5*). Combined, these results support the role of density-dependent competition for resources as a limiting factor for mosquito larval performance. Oviposition assays indicated that female mosquitoes select for larval habitats with conspecifics and that larval density was more important than diet in driving selection for oviposition sites.

**Conclusions:**

This study supports predictions of the P-P hypothesis and provides a mechanistic understanding of the underlying factors driving mosquito oviposition site selection.

## Background

Oviposition-site choice is an essential component of the life history of all mosquito species impacting offspring survival, juvenile development and growth, intra- and inter-specific interactions, predator avoidance and, ultimately, offspring phenotype and fitness [1]. Laboratory and field studies have shown that oviposition site selection by most mosquito species is not random [2-5]. Yet, egg-laying behavior may be complex and highly heterogeneous. This is perhaps because selecting particular habitats is based on several aspects including physical [6] and chemical [7-10] cues, as well as on interspecific and intraspecific density within the habitat [5,11]. Although it is hypothesized that oviposition site selection maximizes offspring fitness by releasing immature stages from predation or competition with other mosquito larvae or organisms over food resources [5,12,13], few studies have empirically estimated the association between mosquito habitat quality, resource competition, and reproductive fitness in mosquitoes [11,14-17].

Though traditionally applied to food foraging behavior, optimal foraging models provide a conceptual framework to consider oviposition behavior as well. The ideal free distribution (IFD) theory postulates that habitats differ in their suitability to support a given species, and that organisms would preferably exploit those habitats that maximize their overall fitness [18]. Moreover, habitat suitability may change over time due to crowding and density-dependence, impacting future patch exploitation dynamics [18]. The IFD theory can be applied to mosquito oviposition site selection under the hypothesis that a female will allocate the majority of her eggs in the most offspring-suitable habitats available until the pressures from conspecific competition become too strong and she is obligated to lay her eggs in the next most suitable habitats (also known as the oviposition preference-offspring performance (P-P) hypothesis [11,15-17,19]). Under the P-P hypothesis, there is a functional relationship between the suitability of an oviposition site, the density of potential competitors and the concentration of food resources.

*Aedes spp.* mosquitoes, as skip ovipositors [20], distribute individual eggs in several oviposition sites rather than as clustered egg rafts [21,22] facilitating experimental evaluation of the P-P hypothesis. Skip oviposition entails high energy consumption for oviposition site detection relative to oviposition of entire clutches in one location, potentially increasing the risk of maternal mortality. Despite this cost, it may reduce sibling competition and ensure greater distribution of progeny, thereby optimizing larval development as well as offspring fitness [20,23]. In this study we tested the P-P hypothesis through two interrelated laboratory experiments using the Asian tiger mosquito, *Aedes albopictus*, as our study system. This species not only feeds on a wide array of hosts (including humans) [24] and is an efficient vector of Chikungunya virus (CHIKV) [25-27], but also has demonstrated the ability to colonize diverse artificial habitats such as bird baths, flowerpots catch basins or rainwater storage containers [27,28]. In the first experiment, we evaluated *Ae. albopictus* larval performance (i.e., mortality rate, time to pupation and time to adult emergence) and fitness in response to two gradients of habitat conditions: diet level and density of conspecific larvae. By recreating those two-factor treatments with the most contrasting outcomes (from the first experiment), the second experiment assessed whether *Ae. albopictus* females select oviposition sites following the predictions of the P-P hypothesis. Given that immature mosquito survival is density-dependent [29-31] and that potential oviposition sites vary in factors like pre-existing conspecific presence and nutrient levels, we predict that a female’s preference for oviposition sites will occur as a tradeoff between both trophic dimensions: habitats with high food content and low intraspecific competition pressure should be preferred as oviposition sites.

## Methods

### Mosquito colonies

All experimental *Ae. albopictus* larvae were F3 obtained from a laboratory colony originated from wild caught eggs collected in Raleigh, NC (Apperson C., personal communication). Mosquito colonies were maintained at 28 °C and 70% relative humidity on a 12:12 (L:D) light schedule. The diet used in this study was a mixture of solid tuna meal, low-heat desiccated, non-defatted solid Argentinian beef liver powder (Now Foods, Bloomingdale, IL), and solid vitamin mix (BioServ, Frenchtown, NJ) mixed at the ratio 2:2:1 respectively (Benedict M.Q., personal communication). The solid mixture was thoroughly mixed in pure water to make a 2% diet aqueous solution for efficient diet-transfer purposes.

### Experiment 1: Assessing performance and fitness over a gradient of habitat conditions

We quantified *Ae. albopictus* immature performance and fitness in artificial containers across gradients of food concentrations (diet) and conspecific densities (CD). Performance was characterized by measuring mortality rate (dead individuals/10), time to pupation (days since egg-hatch), and time to adult emergence (days since egg-hatch). Four diet and four CD levels were considered to generate a four-by-four experimental design with 16 treatments encompassing all possible food-conspecific combinations (Table 1). A total of 10 experimental larvae were exposed to each treatment, keeping experimental larvae separate from the conspecifics by a mesh screen divider (Additional file 1: Figure S1). The experimental larvae were carefully monitored throughout their development until adult emergence, whereas the conspecific larvae were used solely to provide the CD conditions within the container treatments. Each treatment was replicated three times (total of 48 containers) under constant temperature (28%, relative humidity (80%), and light (diel length 12:12 L:D, and with two crepuscular hours mimicking sunrise and sunset illumination) regimes.

**Table 1:** Distribution of treatments per experiment

			**Conspecific density (number of larvae / 300 ml)**			
**Rank**		**5**	**4**	**3**	**2**	**1**
	Daily Diet (mg/300 ml)	0	10	20	40	80
1	0.0 mg	2	2	-	-	2
2	2.0 mg	1, 2	2	1	1	1, 2
3	3.6 mg	1, 2	2	1	1	1, 2
4	7.2 mg	1, 2	2	1	1	1, 2
5	20.0 mg	1	-	1	1	1

Numbers indicate the experiment in which such diet-CD combination was used: 1, Experiment 1 (impact of diet and CD on *Ae. albopictus* performance); 2, Experiment 2 (impact of diet and CD on female oviposition site selection). Diet and CD treatments were ranked in increasing level of quality, and the sum of both ranks used to estimate the container quality index (QI). See methods for more details.

The CD conditions, in ascending order, were: zero larvae, 20 larvae, 40 larvae, and 80 larvae (Table 1). Conspecific larvae were added half as first-instars and half as third-instars to simulate a structured age distribution compatible with field observations as well as to maintain conspecific presence for the duration of the experiment. Diet treatments used in this study included a daily dose of 2.0 mg, 3.6 mg, 7.2 mg, and 20.0 mg of the diet mixture in 300 ml of deionized water.

Larval rearing containers were made out of 473 ml white, plastic, cylindrical food containers (Bauman Paper Co., Lexington, KY) divided by a white mesh screen positioned perpendicularly to the bottom of the container (Additional file 1: Figure S1). The mesh screen was permeable to the diet solution but impermeable to larvae movement. Twenty-four hours prior to the beginning of the experiment, each container was filled with 300 mL of purified water with the appropriate diet mixture to allow bacterial development (a food source for *Ae. albopictus*[32]) and settling of food particles. Following this settling period, 10 F3 generation, recently emerged (~24-hours post hatching) *Ae. albopictus,* I-instar larvae were added to one side of the container (experimental larvae) with conspecific larvae added to the other side of the mesh division at densities described in Table 1. The additions of experimental and conspecific larvae marked the beginning of the experiment.

Experimental larval development was monitored daily by counting the number of molts to each life stage starting from II-instar to pupal stage (after counting, molts were removed from the container). When treatment larvae reached the pupal stage, the pupae were removed from the container (they can be extracted without affecting per-capita food consumption of the remaining larvae). When experimental larvae pupated, the pupae were kept inside the container and the molt(s) removed. Each emerged adult from the experimental larvae was sexed and all emerged females were individually stored at −80**°**C for further wing-length measurements.

Wing length is considered a proxy of female body size and overall mosquito fitness [33,34]. Female adult body size is important to assess fitness because larger females lay more eggs [35,36], thus increasing the probability of successful progeny. Wing-length measurements were conducted for a subset consisting of two randomly selected replicates of each treatment. Wings were separated from the body of the adult females under a Leica MS5 stereozoom microscope (Leica Microsystems Incorporated, Bannockburn, IL). Following dissection, photographs of the wings were taken using a MagnaFire 2.1C CCD camera (Optronics, Goleta, CA) under an Olympus BX60F-3 microscope (Olympus Optical Co., Ltd., Japan) at 4X magnification. Wing length measurements were conducted using the image-processing program ImageJ [37].

### Experiment 2: Empirical estimation of oviposition site preference

Twelve different containers with unique diet-CD combinations were used in single-female oviposition assays (Table 1). As before, half of the conspecific larvae were added as I-instars and half as III-instars. Coffee filter paper was lined along the walls of each container to collect mosquito eggs. The 12 containers were randomly positioned in an enclosed insect tent (75 × 75 × 115 cm; MegaView Science Co., Ltd., Taichung, Taiwan) and left for 48 hours to allow for bacterial growth.

Adult F3 mosquitoes were used for the oviposition assays. Upon adult emergence, a female was placed in a 20x20x20 cm acrylic chamber with male mosquitoes for 24 hours to mate, and provided access to a sedated rabbit on the following day. Use of laboratory animals for blood-feeding mosquitoes was approved by the Centers for Disease Control and Prevention Animal Use Protocols (IACUC protocol # 2245BARRABB). One day after feeding, gravid females were released into the experimental tent containing the array of oviposition sites and left for three days to oviposit the initial batch of eggs. We hypothesized that the initial oviposition would show the strongest correlation with oviposition site selection, given the absence of preexisting eggs, which can act as oviposition attractants [23,38]. Environmental conditions were set as described in the first experiment. Upon completion of each 3-day assay, females were removed from the tents, filter papers were removed from each container and the number of eggs counted. The oviposition trials were replicated four times.

### Data analysis

#### Experiment 1

Daily larval survival under different diet and CD combinations was analyzed using Kaplan-Meier Survival Analysis tests. Mantel-Cox Log-Rank tests were performed to determine whether increases in diet and/or CD significantly affected mosquito survival. Two-way Completely Randomized (CR) Analysis of Variance (ANOVA) tests compared the mean time to pupation and time to adult emergence (log_10_-transformed) across different treatments. The mean of the 10 experimental larvae for both time to pupation and time to emergence was computed for each container before the CR ANOVA. These computed means represented independent observations characterizing each container and thus validated the use of a CR ANOVA as opposed to a repeated/related measures ANOVA [39]. Percent pupation and emergence success rate were highly skewed and thus analyzed using the Kruskal-Wallis test.

A Generalized Additive Mixed Model (GAMM) was applied to determine the association between diet, CD levels and adult mosquito wing length [10,40]. The full model had the form:

$$\begin{array}{ll} Y_{\text{wing}}= & \alpha +f1\left(\text{Diet}\right)+f_{2}\left(CD\right)+f_{3}\left(\text{Diet}∼CD\right)+Zi\\ & +\varepsilon_{j} \end{array}$$

where, $\varepsilon_{j}∼N\left(Ο,\sigma^{2}\right)$. The parameter *Zi* represents a random effects term associated with each observation within a replicate. Given the fairly Normal wing length distribution we used a Gaussian link function to parameterize the model. GAMM models allow for non-linear relationships between the response variable and multiple explanatory variables by incorporating a smoothing function representing the additive component [40]. We fitted by applying a penalized cubic spline function to the data [40]. Four models were developed: one with each variable alone, one model with both variables non-linearly related with each other (expressed as Diet ~ CD) and the full model including all possible combinations of variables. We assessed each model’s ability to fit the data by comparing their Akaike Information Criterion (AIC) scores after a correction for small sample sizes (AICc). A model with ΔAICc = 2 or more units lower than any other model was considered the best, and used to predict the association between wing length, diet and CD. Once the best model was identified, we plotted each predicted *f*_*i*_ as either a curve (single terms) or a surface (diet ~ CD) and assessed the model’s overall fit by reporting pseudo-R^2^ and deviance parameters.

#### Experiment 2

The IFD theory predicts that the number of eggs oviposited will be equally distributed among patches, or cups, with the same intrinsic quality. In habitats that differ in ‘quality’ (measured as the concentration of food and conspecifics), it is predicted that female mosquitoes will deposit eggs in high quality containers first. We developed a container quality index (QI), by first ranking food and CD from low to high and then assigning them values from 1 to 5 (Table 1). By adding the ranked values of food and CD to each treatment we developed the QI index (which ranges from 2 to 10). At the low end of the scale are treatments with no or low diet and high density. The high end of the scale represents treatments with high diet and no or low density (Table 1).

To quantify the association between diet, CD and number of eggs per container a general additive mixed model GAMM was applied. The full model had the form:

$$\begin{array}{ll} Y_{\text{eggs}}= & \alpha +f_{1}\left(\text{Diet}\right)+f_{2}\left(CD\right)+F_{3}\left(\text{Diet}∼CD\right)+Zi\\ & +\varepsilon_{j} \end{array}$$

where, $\varepsilon_{j}∼N\left(Ο,\sigma \right)$. The parameter *Zi* represents a random effects term associated with each observation within a replicate. Given the distribution of eggs per container was skewed (i.e., many containers had no eggs, whereas a few containers had large number of eggs) we used a negative binomial link function to parameterize the model. The GAMM model was tested as before, and included the number of eggs per container on each of the four trials as observations. GAMM models were run in R statistical software’s package mgcv [41]. All other statistical analyses were performed in SPSS 17.0.0 [42].

## Results

### Experiment 1: Assessing performance and fitness over a gradient of habitat conditions

**Figure 1:**
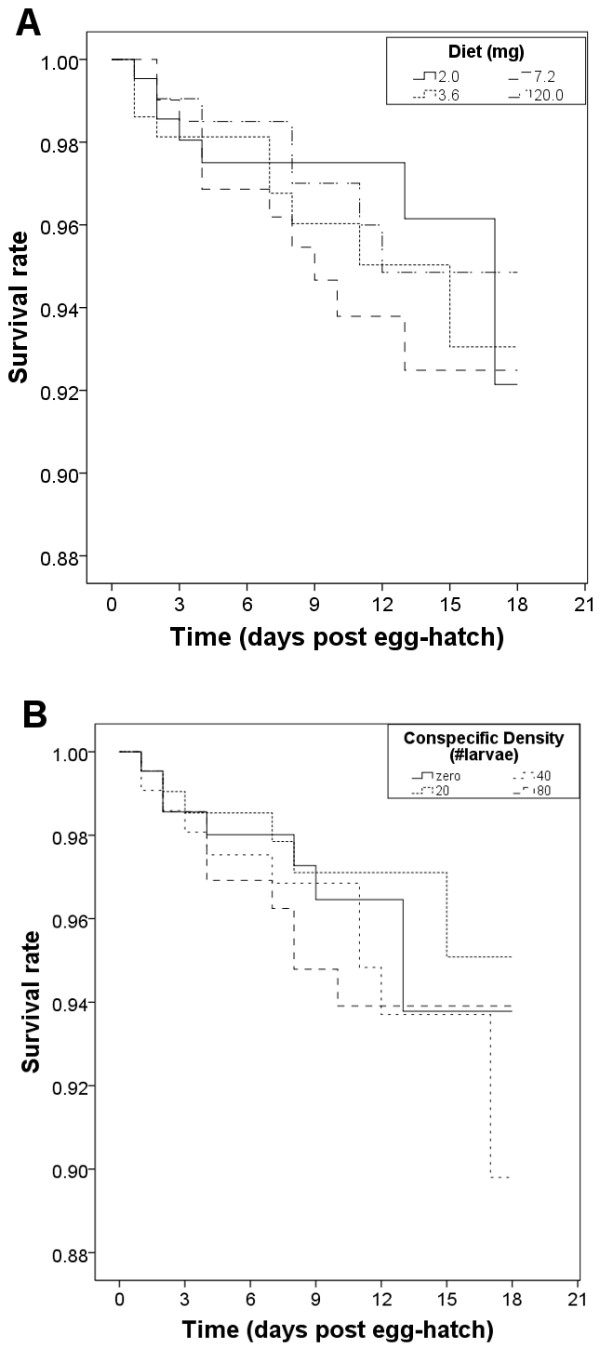
**Impact of diet and CD on larval survival.** (**A**). Survival Analysis Curve using diet (expressed in mg/300 ml) as a potential predictor of mean lifespan (per container type) across all replicates (N = 3). The Kaplan Meier test showed no difference in survival across diet treatments (P = 0.921). (**B**) Survival Analysis Curve using conspecific density (CD) as a potential predictor of mean lifespan (per container type) across all replicates (N = 3). The Kaplan Meier test showed no significant difference in treatments across CD (P = 0.414).

**Figure 2:**
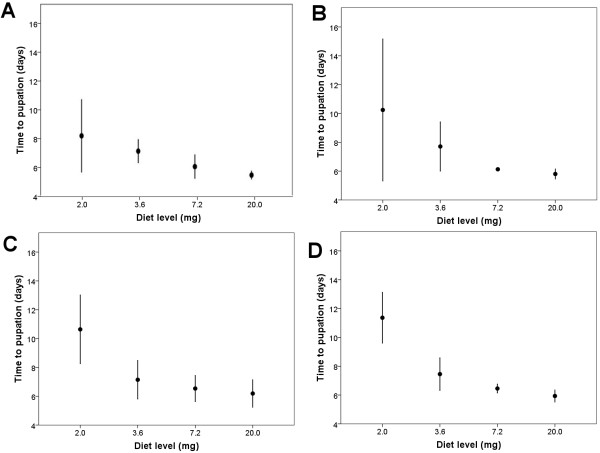
**Development time to larval pupation across ascending diet levels by conspecific density.** (**A**) zero conspecifics. (**B**) 20 conspecifics. (**C**) 40 conspecifics. (**D**) 80 conspecifics. Mean values are indicated in boxes. Diet is indicated in mg/300 ml of water. Each treatment was replicated three times.

Of the 480 experimental larvae, 35 (7.3%) died before emergence. Diet levels alone (i.e., in the absence of conspecific larvae) did not significantly affect immature lifespan (log rank Mantel-Cox, *X*^*2*^ *= 0.492, P > 0.1*) (Figure 1A). Similarly, conspecific density (CD) alone did not significantly affect lifespan per larva (log rank, Mantel-Cox, *X*^*2*^ *= 2.858, P > 0.1*) (Figure 1B). Overall pupation rate was 93.3% and mean time to pupation was 7.4 days (SD ± 1.93 days). The container treatment resulting in experimental larvae with the longest time to pupation was 2.0 mg diet and 80 CD (see Table 1 for full description of treatments), pupating at 11.4 days (SD ± 0.88 days), whereas the treatment with the shortest time to pupation was 20.0 mg and 0 CD, pupating at 5.5 days (SD ± 0.14 days) (Figure 2). Diet and CD (Two-Way CR ANOVA, diet: *F*_*(d.f. =3)*_ *= 57.6, P < 0.001*; CD: *F*_*(3)*_ *= 3.6, P < 0.05*) but not their interaction (*F*_*(9)*_ *= 1.5, P > 0.1*) were significantly associated with time to pupation. Post-Hoc tests indicated that time to pupation was significantly shorter with each increasing diet level except from 7.2 mg to 20.0 mg (Two-Way CR ANOVA Post-Hoc test, *P > 0.1*) (Figure 2, Table 2). In addition, time to pupation significantly increased when CD increased from zero larvae to 80 larvae (Two-Way CR ANOVA Post-Hoc test, *P < 0.05*).

**Table 2:** Association between time to pupation, diet and CD

**Reference treatment**	**Comparisons between levels**		**Mean difference (i-j in days)**	***P***
Diet	2.0 (i)	3.6 (j)	2.7503	< 0.001
		7.2	3.8157	< 0.001
		20.0	4.2607	< 0.001
	3.6	7.2	1.0654	0.032
		20.0	1.5104	0.001
	7.2	20.0	0.4450	1.0
CD	0 (i)	20 (j)	−0.7490	0.261
		40	−0.9051	0.097
		80	−1.0796	0.029
	20	40	−0.1561	1.0
		80	−0.3306	1.0
	40	80	−0.1745	1.0

Results from post-hoc comparisons performed after a Two-Way completely randomized analysis of variance (ANOVA) of time to pupation (days post-egg hatch), diet and conspecific density. Diet is expressed as mg food/300 ml water whereas CD as number of larvae/300 ml water. P-values are expressed after applying a Bonferroni correction for multiple-comparisons.

A total of 445 larvae (92.7%) emerged as adults, comprising 98.0% of the total number of larvae that successfully pupated. Overall, the treatment with the longest time to adult emergence was 2.0 mg and 80 CD, at 13.5 days [SD ± 0.97 days], whereas the treatment with the shortest time to emergence was 20.0 mg and 0 CD, 7.7 days [SD ± 0.10 days]. These trends were qualitatively the same as the ones found for time to pupation (not shown). Diet and CD were both significant predictors of time to emergence (Two-Way CR ANOVA, diet: *F*_*(3)*_ *= 49.0, P < 0.001*; CD: *F*_*(3)*_ *= 3.0, P < 0.05*). The interaction between diet and CD was not significantly associated with the time to emergence (*F*_*(9)*_ *= 0.9, P > 0.1*). Post-Hoc tests indicated that time to emergence was significantly affected with each increasing diet level except from 7.2 mg to 20.0 mg (Two-Way CR ANOVA Post-Hoc test, *P > 0.1*) (Table 3). In addition, time to emergence only increased significantly from CD of zero larvae to 80 larvae (Two-Way ANOVA Post-Hoc test, *P < 0.05*). Increasing CD levels from zero to 40 larvae did not significantly affect development time, suggesting that at least 80 conspecific larvae/300 ml are needed to induce intraspecific crowding and resource competition intense enough to significantly impact larval performance.

**Table 3:** Association between time to emergence, diet and CD Results from post-hoc comparisons performed after a Two-Way completely randomized analysis of variance (ANOVA) of time to emergence (days post-egg hatch), diet and conspecific density

	**Comparisons between levels**		**Mean difference (i-j in days)**	***P***
Diet	2.0 (i)	3.6 (j)	0.0943	< 0.001
		7.2	0.1503	< 0.001
		20.0	0.1790	< 0.001
	3.6	7.2	0.0559	0.008
		20.0	0.0847	< 0.001
	7.2	20.0	00287	0.484
CD	0 (i)	20 (j)	−0.0347	0.235
		40	−0.0363	0.261
		80	−0.440	0.064
	20	40	−0.0016	1.0
		80	−0.0094	1.0
	40	80	−0.0077	1.0

Diet is expressed as mg food/300 ml water whereas CD as number of larvae/300 ml water. P-values are expressed after applying a Bonferroni correction for multiple-comparisons.

**Figure 3:**
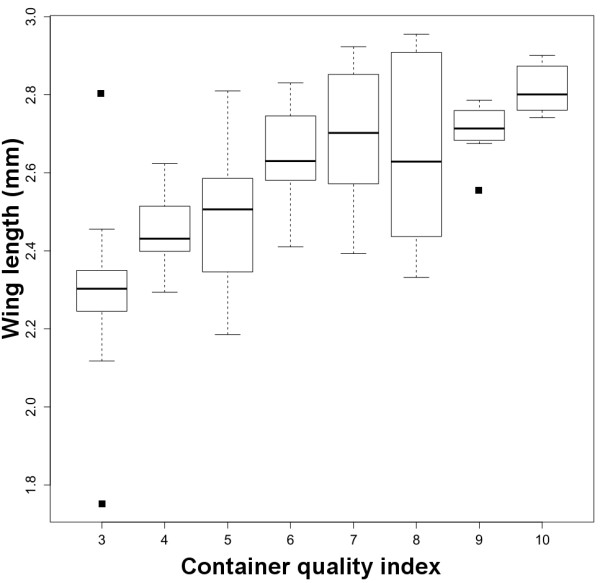
**Association between wing length and the container quality index.** Boxplot indicating median (horizontal line), interquartile (box) and range (dotted lines). Dots represent outlier observations.

**Figure 4:**
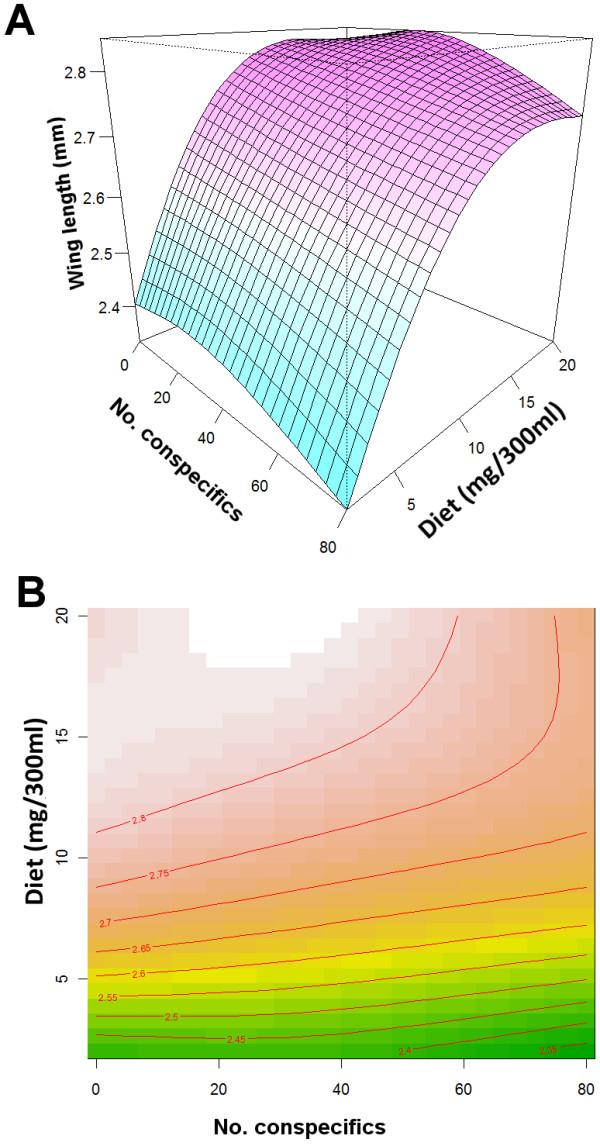
**Association between wing length, diet and CD.** (**A**) Graphic representation of the association between diet, conspecific density and wing length (parameter Diet ~ CD in from the best GAM model). (**B**) Surface representing the association between diet and conspecific density (surface colors) and wing length (red lines). Numbers on top of lines indicate wing length values whereas colors interpolated wing length values.

A total of 131 females were dissected for wing length measurements. Wing length was positively and significantly associated with the container quality index (linear regression coefficient, b = 0.07;SE = 0.007; *P* < 0.001; Figure 3); at higher QI values mosquito fitness was predicted to be highest. The best GAM model (ΔAICc = 7 in comparison to the second best model) included the non-linear association between diet and CD (diet ~ CD) as its sole and significant term (Table 4). This model had an adjusted R^2^ of 0.60 and explained 62% of the deviance. Figure 4A shows the non-linear association between diet, CD, and wing length. Diet had a higher impact on wing length than CD; the number of conspecifics negatively affected wing length only when food levels were low (<5 mg). At intermediate diet levels (>10 mg), increases in CD did not exert any significant change in wing length (Figure 4A). Figure 4B shows the effect of varying diet and CD on adult wing length which, given the association between wing length and reproductive fitness, can be interpreted as an *Ae. albopictus* fitness surface. The diet and CD combination that produced the highest mosquito fitness were >11 mg and < 60 conspecifics/300 ml, respectively (Figure 4B).

**Table 4:** Summary of the best GAMM model estimating the association between wing length, diet and conspecific density

		**Fixed terms**				**Smooth terms**		
**Experiment**	**Parameter**	**Estimate**	**SE**	***Z***	***P***	**Structure**	**DF**	**P**
1	Intercept	2.603	0.018	201	<0.001	f(Diet ~ CD)^*^	8	<0.001
2	Intercept	1.897	0.227	18	<0.001	f(Diet ~ CD)	5	<0.001

* Represents the structure of the smoothing term: f(D) only Diet is significant, f(C) only conspecific density is significant, f(Diet ~ CD) the non-linear association between diet and conspecific density is significant.

### Experiment 2: Empirical estimation of oviposition site preference

Because larval performance (i.e. time to pupation and adult emergence) differed significantly only between diets 2.0, 3.6, and 7.2 mg, these levels plus a level of no food were used as the experimental diets in the oviposition assays. We also concluded in the first experiment that CD only had an effect on larval performance in the range 0 to 80 conspecific larvae. Also, CD only had an effect on fitness (i.e. wing length) when the diet was <5 mg. Therefore, the CD levels used to test oviposition site selection were 0, 10, and 80 conspecific larvae.

**Figure 5:**
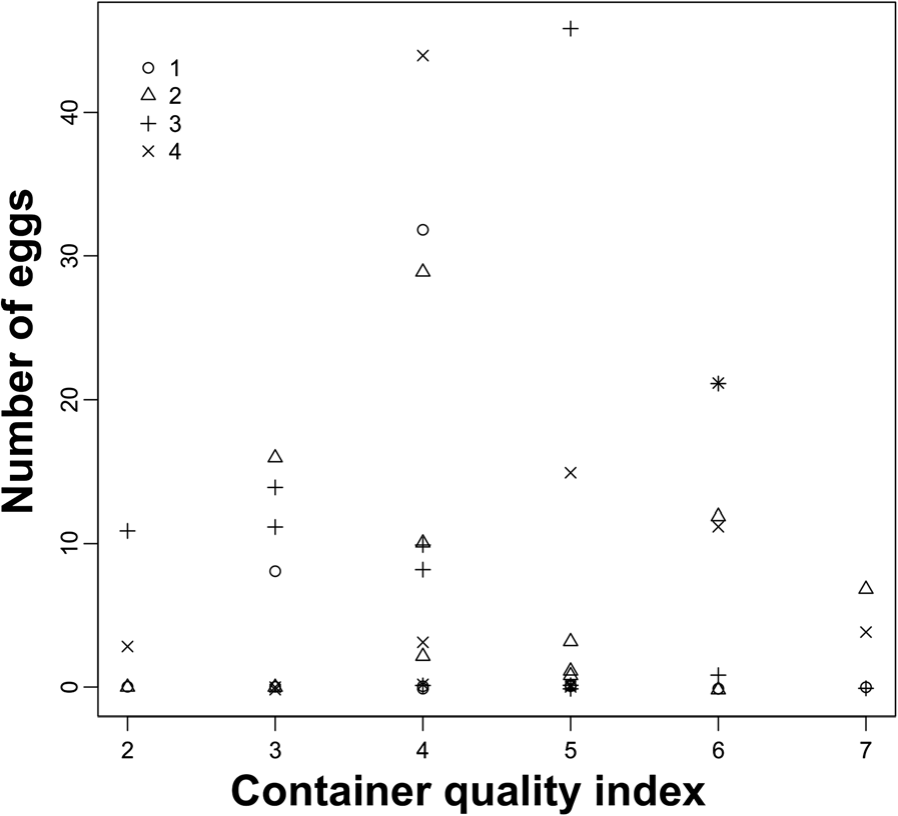
**Results from the oviposition site selection trials.** Association between the number of eggs per container and the container quality index. Each symbol represents a replicate experiment in which a single female was given the choice to oviposit in containers with different combinations of diet and conspecific density (CD) of oviposition sites.

**Figure 6:**
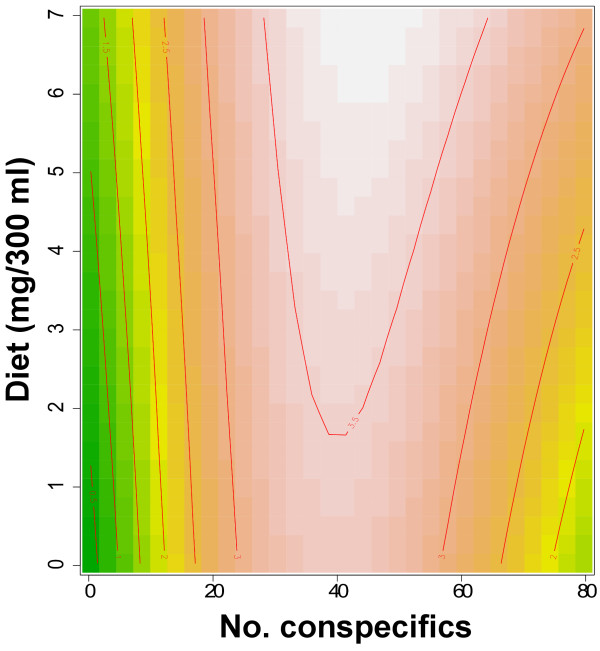
**Association between oviposition, diet and CD.** Surface representing the association between diet and conspecific density and number of laid eggs (surface colors) on a given container (term Diet ~ CD from the best GAM model). Numbers on top of lines indicate the natural logarithm of the number of eggs laid on a given container. Colors indicate interpolated number of eggs per container.

The total numbers of eggs per replicate were: 40, 81, 101 and 122. The observed numbers of eggs laid in each container did not follow a pattern consistent with the expectation of the P-P hypothesis: that increases in the container quality index will be associated with increases in the number of eggs (Figure 5). Instead, most females laid eggs at intermediate container quality indices (Figure 5). Females whose total egg batch was low (~40-80 eggs distributed among all containers), oviposited heavily in containers harboring fewer conspecific larvae (i.e. CD of 10 larvae), whereas females with large egg batches (~100-125 eggs), oviposited preferentially in the high CD containers (i.e. 80 larvae). The best GAM model (ΔAICc = 25) included the non-linear diet ~ CD term as the sole and significant predictor of the number of eggs per container (Table 4, Figure 6). This model had an adjusted R^2^ of 0.1 and explained 23% of the deviance. CD had a larger impact on oviposition site selection than diet (Figure 6); the number of eggs was negatively associated with the number of conspecifics in a container at intermediate (20–50 per 300 ml container) larval densities. Figure 6 shows the importance of the simultaneous consideration of diet and CD when assessing the oviposition preference of a skip ovipositor such as *Ae. albopictus*.

## Discussion

The P-P hypothesis proposes that females should oviposit in suitable habitats to minimize resource-mediated interactions while maximizing offspring fitness [11,15,16,19]. This study confirmed that oviposition site selection by *Ae. albopictus* followed the predictions of the P-P hypothesis for oviposition site selection: 1) females allocated most of their eggs in an array of habitats that conferred high offspring performance and fitness, and 2) conspecific larvae presence and density were the most important factors influencing site selection within a medium to low diet gradient.

Previous studies have described that high diet conditions correlate with shorter *Ae. albopictus* development times [5,31]. In the present study, larval development time decreased with increasing diet level, and such findings were not significantly influenced by the presence of conspecific individuals at medium diet concentrations. The lack of a scaling of development time with diet at high food concentrations indicates that diet only affected larval performance within the range in which competition for food resources operated (i.e., high diets provided more resources than the 10 experimental larvae could compete for) [43], a pattern similarly observed for *Anopheles arabiensis* mosquitoes [44].

Density-dependent competition for food during the early larval stages is considered to be one of the most important factors affecting mosquito population dynamics [31,44,45]. Overcrowding of mosquito larval habitats generally results in retarded growth, high mortality, small and non-uniform sized adults, and decreased fecundity. Although mortality rates differed between treatments, prolonged development time was exclusively observed in high-density treatments (80 conspecific larvae). Such observed density-dependent effect could have resulted from crowding effects (i.e., the direct impact of larger numbers of organisms on access to food and, ultimately, feeding success; [4,5,46]), increased food partitioning (lower food per capita; [46]), or toxins induced by such sources of stress [47]. By separating conspecific from experimental larvae and keeping the experimental larvae at low densities (10 larvae/150 ml of water), this study minimized any possible occurrence of crowding effects focusing on the effect of food partitioning on larval productivity. In our study, the estimated food available per capita (per larva) ranged from 0.00067 mg/mL (2 mg/300 mL per 10 individuals) to 0.0024 mg/mL (7.2 mg/300 mL per 10 individuals), which is comparable to previous studies testing diet gradients ranging from severely limiting to surplus levels [43,44]. High conspecific larval density is associated with high levels of toxin production [47] and depletion of haemolymph glucose levels [38]. Thus, in our study stunting or prolonging development time to the adult stage could have been a product of both resource partition and sub-products of overcrowded larval habitats. By studying the chemical ecology of oviposition sites in association with mosquito population dynamics, future research may be able to further identify the biological processes underlying oviposition site selection by *Ae. albopictus*.

Adult female body size (i.e. quantified as wing length) was measured to estimate maternal fitness resulting from the tested diet and CD conditions. Female fitness is particularly important because larger females lay more eggs, contributing more to a population’s reproductive fitness [35,36]. As predicted, wing length increased proportionally with increases in diet with the highest diet level (20.0 mg) resulting in the longest mean wing length. That the number of conspecific larvae negatively affected wing length only when food levels were low (<5 mg) suggests that shorter development and thus smaller body size outweigh the costs associated with high CD under nutrient limitations. Intense competition (most likely elicited under <11 mg of diet and >60 conspecific larvae [Figure 4B]) may decrease an individual larva’s probability of emergence and survival; therefore, larger body size may need to be sacrificed to ensure adult emergence and thereby maintain (or increase) overall adult population size [48].

In the oviposition assays, females tended to lay eggs in sites where conspecific larvae were present. This finding was counter to expectations following Experiment 1, which concluded that high competition resulting from low food availability negatively affects larval performance and fitness. It would seem advantageous for ovipositing females to avoid sites containing potential competitors to their own progeny. However, the benefit of using conspecifics as a cue of habitat suitability may counteract the potential drawback of conspecific competition [7,49]. Wong *et al*. [50] hypothesized that the presence of *Ae. aegypti* conspecifics may serve as a signal that the site experiences infrequent water turnover and desiccation, and contains adequate food, two critical conditions for larval development. Whether females select oviposition sites due to their adequate larval conditions or other variables naturally found in oviposition sites (e.g. different types of diet, site color, presence of other mosquito and non-mosquito species) remains to be determined.

This study presented several limitations. The estimation of wing measurements from only two replicates affected the fit of the GAM models to the data (as observed in the low deviance explained by the model), which could have been improved with a larger sample size. Also, the CD treatments selected in this study were based on field observations performed in a residential neighborhood of Atlanta, GA, (MY unpublished data) and may not necessarily reflect the full range of naturally-occurring CDs in which *Ae. albopictus* larvae could develop. Also, the use of a water-based food formulation instead reduced the amount of debris and homogenized the color of the water between containers. By performing single female assays we were able to easily quantify oviposition site selection. However, the inclusion of more than one female could have provided additional insights about the role of female density on oviposition site selection. It is unknown whether the presence of other gravid females can act as an additional oviposition cue, and such behavioral mechanisms deserve further investigation.

## Conclusions

Current control strategies have shown transient or no perceivable impacts in *Ae. albopictus* populations, and point to the need for a better understanding of the biological and ecological factors regulating natural populations [27]. Our findings expand current knowledge on the ecology of *Ae. albopictus* by showing that conspecific density may be an important driver in oviposition site selection. Further understanding of the mechanisms facilitating successful *Ae. albopictus* oviposition behavior will aid in the development of improved control strategies, particularly at the reproductive level.

## Competing interests

The authors declare that they have no competing interests.

## Authors’ contributions

All authors participated in the conceptualization of the study. MY, FK, JC and JM conducted laboratory experiments. MY and GVP analyzed the data. MY, and GVP drafted the manuscript. All authors participated in the revision of the manuscript and approved the submitted version. All authors read and approved the final manuscript.

## Supplementary Material

Additional file 1 **Figure S1.**Experimental setup. (A) Plastic 16-ounce food container (Bauman Paper Company, Lexington, KY) divided by a white mesh screen positioned perpendicularly to the bottom of the container. The mesh screen was permeable enough to allow the food mixture to flow homogeneously throughout the container but impermeable enough to keep pre-existing conspecific larvae (“C” side of cup) separate from the experimental larvae (“E” side of cup). (B) Upon pupation within a container, the top of the container was covered with a mesh screen to prevent emerging adults from escaping. The small opening in the mesh screen covered by a cotton ball allowed easy extraction of adults upon emergence. Click here for file
