# Supplementary material for: Diet and density dependent competition affect larval performance and oviposition site selection in the mosquito species Aedes albopictus (Diptera: Culicidae)
Source: Parasit Vectors. 2012 Oct 8;5:225. doi: 10.1186/1756-3305-5-225 (PMC3481443; doi:10.1186/1756-3305-5-225)

Figure S1. Plastic 16-ounce food container (Bauman Paper Company, Lexington, KY) divided by a white mesh screen positioned perpendicularly to the bottom of the container. The mesh screen was permeable enough to allow the beef mixture solution to flow homogeneously throughout the container but impermeable enough to keep pre-existing conspecific larvae (“C” side of cup) separate from the experimental larvae (“E” side of cup), B) upon pupation within a container, the top of the container was covered with a mesh screen to prevent emerging adults from escaping. The small opening in the mesh screen covered by a cotton ball allowed easy extraction of adults upon emergence.

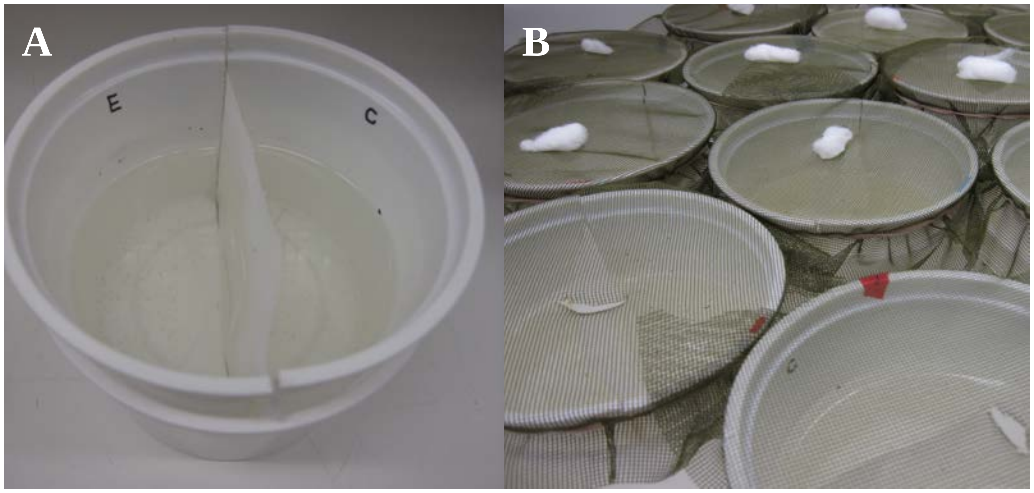

Supplement: Additional file 1 — Figure S1.Experimental setup. (A) Plastic 16-ounce food container (Bauman Paper Company, Lexington, KY) divided by a white mesh screen positioned perpendicularly to the bottom of the container. The mesh screen was permeable enough to allow the food mixture to flow homogeneously throughout the container but impermeable enough to keep pre-existing conspecific larvae (“C” side of cup) separate from the experimental larvae (“E” side of cup). (B) Upon pupation within a container, the top of the container was covered with a mesh screen to prevent emerging adults from escaping. The small opening in the mesh screen covered by a cotton ball allowed easy extraction of adults upon emergence. [file 1756-3305-5-225-S1.pdf]
